# Emergence of rifampin-resistant staphylococci after rifaximin administration in cirrhotic patients

**DOI:** 10.1371/journal.pone.0186120

**Published:** 2017-10-05

**Authors:** Ji Young Chang, Seong-Eun Kim, Tae Hun Kim, So-Youn Woo, Min Sun Ryu, Yang-Hee Joo, Ko Eun Lee, Jihyun Lee, Kang Hoon Lee, Chang Mo Moon, Hye-Kyung Jung, Ki-Nam Shim, Sung-Ae Jung

**Affiliations:** 1 Departments of Internal Medicine, Ewha Womans University College of Medicine, Seoul, Korea; 2 Departments of Microbiology, Ewha Womans University College of Medicine, Seoul, Korea; 3 Departments of Ewha Medical Institute, Ewha Womans University College of Medicine, Seoul, Korea; Universitatsklinikum Munster, GERMANY

## Abstract

**Objectives:**

Rifaximin, a poorly absorbed antibiotics, has gut-specific therapeutic effects. Although frequently prescribed to manipulate intestinal luminal bacterial population in various diseases, the possible induction of antibacterial cross-resistance to a target pathogen is a major concern in long-term rifaximin administration. We aimed to evaluate whether rifampin-resistant staphylococci could evolve after rifaximin treatment in cirrhotic patients.

**Method:**

A total of 25 cirrhotic patients who were administered rifaximin for the prevention of hepatic encephalopathy were enrolled. Swabs from both hands and the perianal skin were acquired on day 0 (before rifaximin treatment), period 1 (1–7 weeks after treatment), and period 2 (8–16 weeks after treatment) the staphylococcal strain identification and rifampin-resistance testing.

**Results:**

A total of 198 staphylococcal isolates from 15 species were identified. *Staphylococcus epidermidis* was isolated most frequently, and *Staphylococcus haemolyticus* was the most common resistant species both from hands and perianal skin. Eleven patients (44.0%) developed rifampin-resistant staphylococcal isolates in period 1. Among these patients, only six (54.5%) were found to have rifampin-resistant isolates in period 2, with no significant infectious events. Rifampin-resistant staphylococcal isolates were more frequently found in perianal skin than from the hands. No patients acquired a newly resistant strain in period 2.

**Conclusions:**

About one-half of cirrhotic patients in this study developed rifampin-resistant staphylococcal isolates after rifaximin treatment. Although the resistant strains were no longer detected in about half of the patients in the short-term, the long-term influence of this drug treatment should be determined.

## Introduction

Rifaximin, belonging to the rifamycin group of antibiotics, has broad-spectrum antibacterial activity against gram-positive, and gram-negative bacteria, and also against aerobic and anaerobic microorganisms [1, 2]. Rifaximin has high availability after oral intake but is poorly absorbed into systemic circulation, resulting in a high concentration in the gastrointestinal lumen, and 97% of the drug is excreted through feces in an unchanged form [3, 4]. These unique properties render rifaximin a gut-specific therapeutic agent, which is therefore widely used for small intestinal bacterial overgrowth, traveler’s diarrhea, hepatic encephalopathy, and colonic diverticular disease [5–7]. Additionally, rifaximin was recently approved for the treatment of diarrhea-predominant irritable bowel syndrome (IBS-D) by the US Food and Drug Administration (FDA) [8].

Along with increased use and the expanded indications of rifaximin, emerging bacterial cross-resistance to other antibiotics has become a major concern. Cross-resistance to the structurally similar antibiotic rifampicin, which is used for the treatment of tuberculosis, meningococcal disease, and staphylococcal foreign body infection [9] has been documented [10]. However, results are inconsistent regarding the influence of rifaximin administration for the development of cross-resistance to rifampin in real clinical settings. The development of cross-resistance to rifampin after rifaximin administration has been confirmed in staphylococci, while this cross-resistance has not been identified in other microorganisms such as *Escherichia coli* (*E*. *coli*), *Mycobacterium tuberculosis* (*M*. *tuberculosis*), or *Clostridium difficile (C*. *difficile)*. Rifampin-resistant staphylococci after rifaximin administration have been found in healthy adults [10], but this cross-resistance has not been properly evaluated in patients who need rifaximin as a therapeutic intervention.

A recent study evaluated the efficacy of repeated rifaximin treatment in patients with relapsing IBS-D symptoms who had responded to an initial 2-week course of rifaximin treatment. The safety of rifaximin administration was demonstrated for several months of treatment [11]; however, this study excluded patients who had significant medical comorbidities.

Rifaximin is approved for the prevention of hepatic encephalopathy in liver cirrhosis patients [12], and this treatment shows long-term reduction in hepatic encephalopathy-related and all-cause hospitalization [13]. For the effective prevention of hepatic encephalopathy in advanced cirrhotic patients, long-term administration of rifaximin is inevitable. Consequently, the development of bacterial cross-resistance has become a major concern.

In this prospective study, we evaluated how frequently rifampin-resistant staphylococci emerge after rifaximin treatment and examined the clinical progress of colonization after rifaximin treatment in cirrhotic patients.

## Materials and methods

### Patient enrollment

Eligible subjects were liver cirrhotic patients who were prescribed rifaximin for the prevention of hepatic encephalopathy. Patients who agreed to participate in the study by giving informed written consent were enrolled. A total of 38 liver cirrhotic patients were included from March 2015 to April 2016 in Ewha Womans University Mokdong Hospital, Seoul, Korea. The patients took rifaximin (Normix^®^, Alfa Wassermann) 400 mg tid orally according to the hepatologist prescription, and rifaximin was discontinued if the patients were found to have rifaximin-resistant staphylococcus during this study and were also confirmed with low risk of ongoing hepatic encephalopathy. Swabs from both hands and perianal skin were acquired on day 0 (before rifaximin treatment), period 1 (between 1–7 weeks), and period 2 (between 8–16 weeks).

Patients who had a history of rifaximin or rifamycin group antibiotic administration or a history of specific disease requiring rifampin treatment including tuberculosis, meningococcal infection, and foreign body infection were excluded from this study. Patients who initially had rifampin-resistant staphylococcal isolates were also excluded. Medical records of all enrolled patients were reviewed, and the patients’ clinical course was followed until December 2016. The Institutional Review Board of Ewha Womans University Hospital approved this study (IRB number; 2014-06-026-008).

### Staphylococcus identification and rifampin susceptibility testing

Swabs from the hands and perianal skin were cultured on blood agar plates for 24–72 hours at 35°C. Colonies with appropriate staphylococcal morphology were sub-cultured on a subsequent blood agar plate, followed by gram staining and catalase tests. Staphylococcal species were confirmed if the colonies were both gram- and catalase-positive. The API Staph (BIOMÉRIEUX, Marcy-I’Etoile, France) kit was used for identification of staphylococcal genera. All isolated staphylococci were tested for rifampin susceptibility by measuring the minimum inhibitory concentration (MIC) using the E-test Rifampicin strip (BIOMÉRIEUX, Marcy-I’Etoile, France).

Bacteria capable of growth at an MIC ≥ 4 μg/mL were considered rifampin-resistant, and those with an MIC ≤ 1 μg/mL were considered susceptible by the CLSI (Clinical and Laboratory Standards Institute) method [10].

### Statistical analysis

All statistical analyses were performed using the SPSS program, version 22.0. Continuous variables are presented as the mean with standard deviation, and categorical variables are presented as number with percentage. To evaluate the predictive factors associated with persisting detection of rifampin-resistant staphylococcus in period 2 among the patients with rifampin-resistant staphylococcus in period 1, Student’s *t-*test was used for continuous variables, and the chi-square test or Fisher’s exact test was used for categorical variables. *P* values < 0.05 were considered statistically significant.

## Results

### Clinical characteristics of enrolled cirrhotic patients

**Fig 1:**
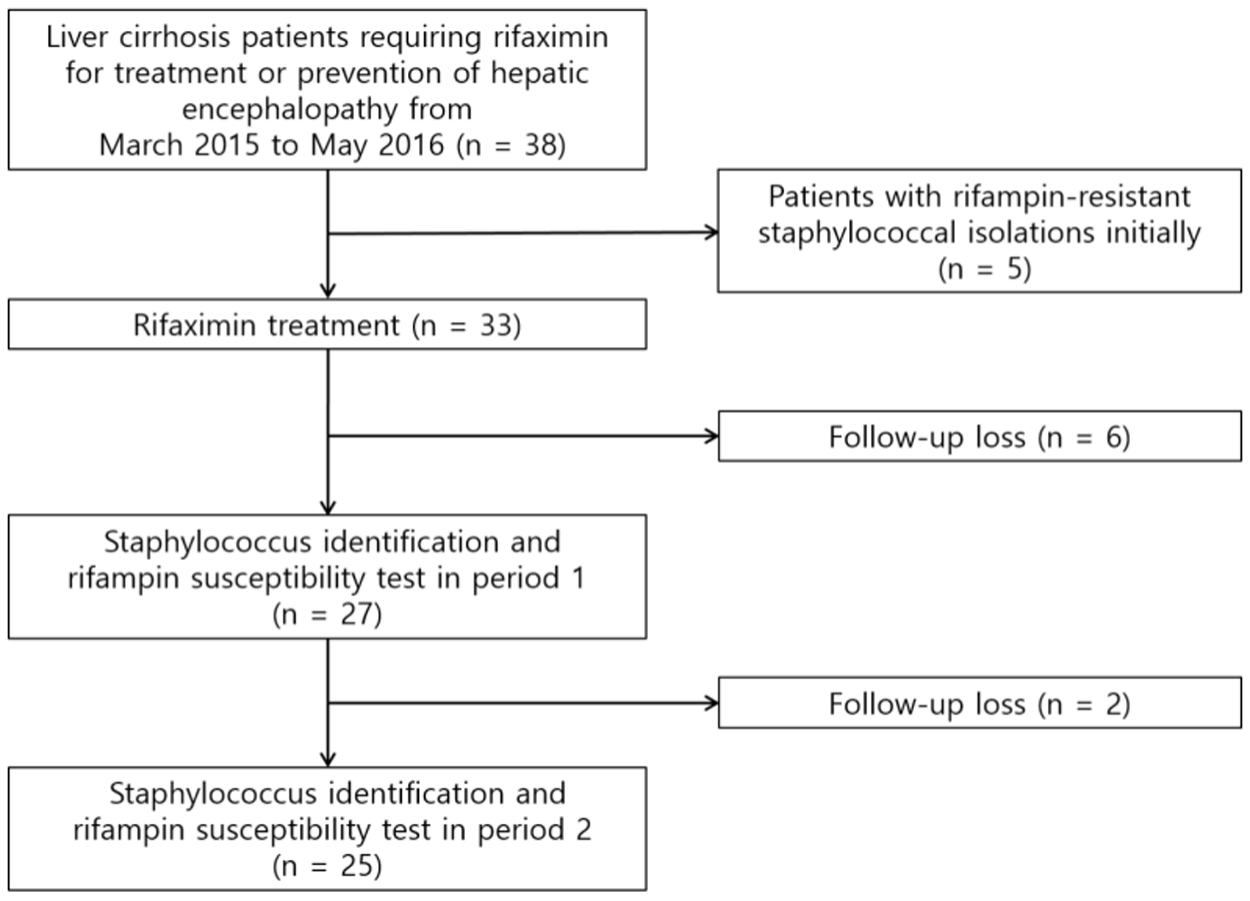
Flow chart of the study design.

Of the 38 cirrhotic patients, five initially demonstrated rifampin-resistant staphylococcal isolates, and eight who did not follow the assigned schedule were excluded. Thus, the data from 25 patients who completed the study were finally analyzed. A detailed flow chart is shown in Fig 1.

The mean patients’ age was 62.2 years, with 80.0% male patients. The most common cause of liver cirrhosis was alcohol use (68.0%), followed by viral infection (28.0%) and a cryptogenic cause (4.0%). Patients were classified as Child A (N = 16; 64%), B (N = 7; 28%), or C (N = 2; 8%). Nine patients (36.0%) were hospitalized at the time of rifaximin treatment initiation, and 6 (24.0%) patients had a history of other antibiotic treatment within 3 months of rifaximin administration. The mean duration of rifaximin treatment was 16.8 days (Table 1). There were no significant drug-related adverse events during this study.

**Table 1:** Baseline characteristics of the enrolled patients.

Age, mean ± SD (years)	62.2 ± 8.4
Male (%)	20 (80.0)
Comorbidities (%)	
Hypertension	5 (20.0)
Diabetes mellitus	6 (24.0)
Alcohol consumption (%)	14 (56.0)
Smoking habit (%)	6 (24.0)
Etiology of liver cirrhosis (%)	
Viral	7 (28.0)
Alcohol	17 (68.0)
Cryptogenic	1 (4.0)
Child score (%)	
A	16 (64.0)
B	7 (28.0)
C	2 (8.0)
Past admission history (%)	
Yes	15 (60.0)
No	10 (40.0)
History of other antibiotics (%)	6 (24.0)
Hospitalized at the time of treatment initiation (%)	9 (36.0)
Duration of rifaximin, mean ± SD (days)	16.8 ± 11.9

SD, standard deviation

### Identification of rifampin-resistant staphylococcal isolates

During the study period, a total of 198 staphylococcal isolates from 15 species were identified. *Staphylococcus epidermidis* (*S*. *epidermidis*) was the most frequently isolated strain, and *Staphylococcus haemolyticus* (*S*. *haemolyticus*) was the most common rifampin-resistant strain isolated from both hands and perianal skin (Table 2).

**Table 2:** Staphylococcal species isolated during the study.

Species	Hand (total/resistant)	Perianal (total/resistant)
*Staphylococcus epidermidis*	43/3	19/4
*Staphylococcus aureus*	18/1	13/6
*Staphylococcus haemolyticus*	17/7	15/9
*Staphylococcus capitis*	12/1	2/0
*Staphylococcus warneri*	12/0	2/0
*Staphylococcus hominis*	8/0	3/0
*Staphylococcus xylosus*	5/1	5/1
*Staphylococcus lugdunensis*	5/0	6/0
*Staphylococcus caprae*	5/0	0
*Staphylococcus chromogenes*	1/0	2/0
*Staphylococcus saprophyticus*	1/0	0
*Staphylococcus simulans*	1/0	0
*Staphylococcus cohnii cohnii*	1/0	0
*Staphylococcus sciuri*	0	2/0
*Staphylococcus lentus*	0	1/1
Total	128/13	70/21

Before rifaximin treatment, staphylococcal colonization was detected in all enrolled patients, with 43 rifampin-sensitive staphylococcal isolates identified: 32 isolated from hands (24 patients) and the other 11 isolates from the perianal skin (15 patients). Eighteen rifampin-resistant staphylococcal isolates from 11 patients (44.0%, 11/25) developed in period 1, 7 isolates from hands (6 patients) and 11 isolates from the perianal skin (10 patients). The median MIC of these isolates was 32.0 (interquartile range (IQR), 4.0–32.0) μg/mL. Seven of the above 11 patients (63.6%) developed resistant isolates within 3 weeks of initiation of rifaximin treatment. The first location of rifampin-resistant isolates was the perianal skin in 5 patients (45.4%), both perianal skin and hands in 5 patients (45.4%), and on hands only in 1 patient (9.1%). Seven rifampin-resistant isolates from hands (3 patients) and 10 isolates from perianal skin (6 patients) were identified in period 2. These isolates showed a median MIC of 32.0 (IQR, 4.0–32.0) μg/mL. No new resistant strains were acquired in period 2. Rifampin-resistant staphylococcal isolates were more frequently found from the perianal skin than the skin of hands in both periods 1 and 2 (Table 3).

**Table 3:** Incidence of staphylococcal isolates during the study.

	Period 0	Period 1	Period 2
Patients with sensitive isolates	25	14	19
From hand	24	14	18
From perianal skin	15	10	14
Patients with resistant isolates	-	11*	6*
From hand	-	6	3
From perianal skin	-	10	6
Staphylococcal isolates	43	73	89
Rifampin-sensitive isolates	43	55	72
From hand	32	40	48
From perianal skin	11	15	24
Rifampin-resistant isolates	-	18	17
From hand	-	7	7
From perianal skin	-	11	10

*Among the 11 patients with rifampin-resistant isolates in period 1, six were found to have rifampin-resistant isolates in period 2.

### Clinical characteristics of patients with rifampin-resistant staphylococci

Clinical data from the 11 patients with rifampin-resistant staphylococci in period 1 were reviewed. Eight patients (50.0%, 8/16) of Child A, 2 patients (28.6%, 2/7) of Child B, and 1 (50.0%, 1/2) patient of Child C grade showed rifampin-resistant staphylococcal isolates during period 1.

Of the 11 patients who developed rifampin-resistant isolates in period 1, six (54.5%) were found to have consistently rifampin-resistant isolates during period 2, whereas the resistant strains were no longer detected in the other patients (45.5%). There was no significant difference in clinical characteristics between the patients who still showed rifampin-resistant staphylococcus colonization and the patients in whom rifampin-resistant staphylococci were no longer detected (Table 4).

**Table 4:** Predictive factors for persisting detection of rifampin-resistant staphylococcus in period 2 among the patients with rifampin-resistant staphylococcus in period 1.

	Persisting detection of rifampin-resistant staphylococcus in period 2	No detection of rifampin-resistant staphylococcus in period 2	*P* value
Age, mean ± SD (years)	67.0 ± 9.49	61.4 ± 4.34	0.257
Male (%)	5 (83.3)	3 (60.6)	0.545
Comorbidities (%)			
Hypertension	1 (16.7)	1 (20.0)	> 0.999
Diabetes mellitus	1 (16.7)	1 (20.0)	> 0.999
Alcohol consumption (%)	3 (50.0)	1 (20.0)	0.545
Smoking habit (%)	1 (16.7)	1 (20.0)	> 0.999
Etiology of liver cirrhosis (%)			0.545
Viral	3 (50.0)	1 (20.0)	
Alcohol	3 (50.0)	3 (60.0)	
Cryptogenic	0 (0)	1 (20.0)	
Child score (%)			0.727
A	4 (66.7)	4 (80.0)	
B	1 (16.7)	1 (20.0)	
C	1 (16.7)	0 (0)	
Past admission history (%)	5 (83.3)	3 (60.0)	0.545
History of other antibiotics (%)	2 (33.3)	2 (40.0)	> 0.999
Admission at the time of treatment (%)	2 (40.0)	2 (33.3)	> 0.999
Duration of rifaximin, mean ± SD (days)	19.67 ± 20.12	19.40 ± 10.21	0.979
Interval for development of rifaximin resistant staphylococcus in period 1, mean ± SD (days)	2.00 ± 1.41	1.20 ± 1.30	0.359
Development of infectious disease (%)	3 (50.0)	0 (0)	0.182

SD, standard deviation

Table 5 demonstrates the detailed changes in strains of rifampin-resistant staphylococcal isolates from period 1 to period 2. Among the 6 patients who showed continued rifampin-resistant staphylococcal isolates in period 2, five harbored different species of rifampin-resistant staphylococcus from those in period 1.

**Table 5:** Identification of rifampin-resistant staphylococci in each period.

	Period 1	Period 2
**Patient 1**		
Hand	*S*. *epidermidis*, *S*. *xylosus*	*S*. *epidermidis*, *S*. *hominis*
Perianal skin	*S*. *aureus*	*S*. *epidermidis*
**Patient 2**		
Hand	*S*. *hominis*	*S*. *capitis*, *S*. *epidermidis*
Perianal skin	*S*. *hominis*, *S*. *xylosus*	*S*. *hominis*
**Patient 3**		
Hand	**-**	**-**
Perianal skin	*S*. *lentus*	**-**
**Patient 4**		
Hand	**-**	*S*. *aureus*, *S*. *hominis*
Perianal skin	*S*. *hominis*	*S*. *aureus*
**Patient 5**		
Hand	*S*. *hominis*	**-**
Perianal skin	*S*. *aureus*	**-**
**Patient 6**		
Hand	*S*. *hominis*	**-**
Perianal skin	*S*. *hominis*	**-**
**Patient 7**		
Hand	*S*. *hominis*	**-**
Perianal skin	*S*. *hominis*	*S*. *hominis*
**Patient 8**		
Hand	*S*. *hominis*	**-**
Perianal skin	**-**	**-**
**Patient 9**		
Hand	**-**	**-**
Perianal skin	*S*. *epidermidis*	*S*. *aureus*, *S*. *epidermidis*
**Patient 10**		
Hand	**-**	**-**
Perianal skin	*S*. *epidermidis*	*S*. *hominis*
**Patient 11**		
Hand	**-**	**-**
Perianal skin	*S*. *aureus*	**-**

There were 6 patients who had history of other antibiotic intake before rifaximin treatment, and half of them showed rifampin-resistant staphylococcal isolates in period 1. During the follow-up period (variable up to 15 months) after rifaximin treatment, infectious diseases developed in 5 patients: 2 patients with pneumonia and 3 patients with catheter-related sepsis, liver abscess, and enterocolitis, respectively; two of them had history of other antibiotic intake. Rifampin-resistant strains were not identified in any of these infected patients. The patient who developed catheter-related sepsis after discontinuation of rifaximin was treated with third-generation cephalosporin for 8 days, and the pathogen was identified as rifampin-sensitive *S*. *epidermidis*. Rifampin-sensitive *S*. *haemolyticus* was identified in the ascites of the patient who developed bacterial peritonitis after 9.8 months of rifaximin discontinuation, and he had no history of other antibiotic intake. The other 3 patients had non-staphylococcal associated infectious disease.

## Discussion

This study is the first to analyze rifampin resistance after rifaximin intake in a real clinical setting. The aim of our study was to evaluate whether rifampin-resistant staphylococci could develop after rifaximin treatment in immunocompromised patients and whether the emergence and colonization of the bacteria could influence patients’ clinical course. In Korea, rifaximin was approved for the treatment and prevention of hepatic encephalopathy in addition to other gastrointestinal infections; therefore, we analyzed cirrhotic patients in this study.

All enrolled patients were found to have staphylococcal colonization, and 44.0% of patients developed rifampin-resistant staphylococcal isolates after rifaximin treatment. The rifampin-resistant staphylococcal isolates emerged within 3 weeks after rifaximin treatment in this study group. The fastest rifampin-resistant staphylococcal isolate was found on day 10 of rifaximin intake. About half of the patients (54.5%) were found to maintain colonization with rifampin-resistant isolates after discontinuation of rifaximin, without acquisition of a new resistant strain in period 2. The resistant strains were no longer detected in the 63.6% of patients after discontinuation of rifaximin. In patients with resistant strains, there was no correlation between duration of rifaximin treatment and time to culture conversion (negative culture result of rifampin-resistant staphylococcus). Rifampin-resistant staphylococcal isolates were more frequently found from the perianal skin than from hand skin. The median MIC of detected rifampin-resistant isolates was 32.0 (IQR, 4.0–32.0) μg/mL in both periods 1 and 2. When EUCAST (European Committee on Antimicrobial Susceptibility Testing) was used, in which rifampin MIC > 0.5 μg/mL was considered resistant, and MIC ≤ 0.06 μg/mL was considered susceptible to rifampin [14], the percentage of rifampin-resistant isolates was higher than that determined by CLSI in periods 1 (27.4%, 20/73) and 2 (21.3%, 19/89), but these changes were negligible, producing no change in MIC distribution.

*S*. *epidermidis* is the most common bacterial species isolated from human skin, found on all body parts [15]. *S*. *aureus* is another common species and is the most eminent pathogen of human infection among staphylococci [15]. The rate of *S*. *aureus* colonization on the hands is known to be 21% in the healthy population [16], while persistent and transient colonization is reported at 20% and 30%, respectively [15]. In our study, *S*. *epidermidis* and *S*. *aureus* were the most commonly found species, but the rate of staphylococcal colonization was higher than in the healthy population.

Rifaximin, a semisynthetic derivative of rifamycin has broad spectrum activity against aerobic and anaerobic gram-negative and gram-positive microorganisms [1, 2]. The mechanism of rifaximin antimicrobial activity is inhibition of RNA synthesis by binding with the β-subunit of the bacterial DNA-dependent RNA polymerase [17]. Rifaximin is not affected by gastric fluid [1], and its additional pyridoimidazole ring minimizes systemic absorption, enabling high concentrations of the drug to remain in the gastrointestinal tract [18]. Nearly 97% of rifaximin is excreted in the stool in unchanged form, with less than 0.4% bioavailability in the blood after oral administration [3, 4]; thus, there is no need for dose adjustments for patients with hepatic dysfunction or renal insufficiency [1]. The adverse effects of rifaximin are also relatively tolerable, and it has a low level of interaction with other drugs sharing the cytochrome P-450 metabolism pathway [19].

By virtue of these unique rifaximin properties–including broad spectrum bacterial coverage, easy availability for oral intake, gut-specific therapeutic effects arising from high gut concentrations, and safety–the clinical indications have been expanded from treatment of traveler’s diarrhea (May 25, 2004) [12] to prevention of hepatic encephalopathy (March 25, 2010) [12] and treatment for irritable bowel syndrome with diarrhea (May 27, 2015) by the US-FDA [8]. Other possible indications are also considered for infectious diarrhea, inflammatory bowel disease, small bowel bacterial overgrowth, and non-complicated diverticular disease [1].

With the broad use of rifaximin, many researchers are concerned about the possibility of antimicrobial resistance. The mechanism for bacteria to obtain rifamycin resistance is associated with DNA-dependent RNA polymerase encoded in the *rpoB* gene, and specific mutations in this gene are found to lead to cross-resistance to other rifamycin derivatives which are turned out in *in vitro* experiments with *S*. *aureus* [20], *M*. *tuberculosis* [21, 22], and *C*. *difficile* [23]. Specific point mutations could result in resistance to all rifamycins or only some of them [21]. Also, activity of bacterial multidrug resistance (MDR) efflux pump systems, which lower the intracellular drug concentration, is another possible mechanism of resistance [24].

Several studies have reported a low possibility of development of rifaximin antimicrobial resistance. In vitro and animal studies reported that rifaximin plasma concentration was not sufficient to develop rifaximin resistance or cross-resistance to rifampin in *M*. *tuberculosis* [25, 26]. Regarding the effect of rifaximin on gut flora and staphylococcal resistance, an in vivo animal study reported that rifaximin could reduce the total bacterial count in the small intestine; however, recovery of bacterial counts occurred a few days after cessation of therapy [27]. The authors of this study conclude no influence of rifaximin on the development of rifampin-resistance by demonstrating no increase of rifampin-resistant staphylococci after rifaximin treatment using rifampin susceptibility tests [27].

On the other hand, opposite results of spontaneous rifaximin-resistant mutants were reported [28], and rifampin-resistant *C*. *difficile* infection associated with previous exposure to rifamycin was demonstrated based on patients hospitalized for the treatment of *C*. *difficile* infection [29]. Staphylococcal isolates with cross-resistance to rifampin were detected on the skin of healthy volunteers after oral intake of rifaximin. The authors of the study assume that contact between stool containing rifaximin and perianal staphylococcus was the mode of acquisition of rifampin-resistance because all of the resistant strains first appeared on the perianal skin [10]. They also suggest the clinical relevance of cross-resistance between rifaximin and rifampin by reporting a case of bacteremia with rifampin-resistant *S*. *aureus* after receiving rifaximin [30]. Especially, rifampin has a pivotal role in treatment of methicillin-resistant *S*. *aureus* (MRSA)-associated foreign body infection as an antibiofilm agent [31]; thus, rifampin resistance could result in fatal treatment failure. According to a recent Korean multi-center study in which our hospital participated, both community-associated MRSA and healthcare-associated MRSA isolates showed high susceptibility to rifampin, nearly 95% [32]. However, a Spanish study reported that the prevalence of rifampin-resistance was higher in MRSA than in methicillin-sensitive *S*. *aureus* although the prevalence of rifampin-resistance itself was low among *S*. *aureus* isolates (0.59%) [33]. It is impressive that all rifampin-resistant *S*. *aureus* isolated from clinical specimens were MRSA in a China study [34]. Therefore, co-existence of methicillin resistance in rifampin-resistant staphylococcus needs to be investigated, though we could not perform such an analysis in this study.

In accordance with the above studies, our study demonstrates cross-resistance between rifaximin and rifampin in staphylococci. Although not all resistant strains were detected on the perianal skin in period 1, about 91% of the resistant strains were detected on either the perianal skin or both the perianal skin and the hands, which strengthens the suggested mechanism of acquired rifampin-resistance in staphylococci. We also showed that this resistance can develop rapidly within 2 weeks and can be detected until 22 weeks after rifaximin discontinuation. We did not find any significant clinical factors associated with persisting detection/or lack of detection of rifampin-resistant staphylococci in period 2 in the patients with rifampin-resistant staphylococcus in period 1. This might imply that there is no predictive factor in deciding which patients should be followed up to confirm whether rifampin-resistant staphylococci will persist or disappear. Fortunately, although the emergence of the resistant strains was identified, no infectious diseases related to rifampin-resistant staphylococci including MRSA infection were found in the patients during the 15-month follow-up period.

In our study, 5 patients who had no history of previous rifaximin or rifampin intake were found to have rifampin-resistant staphylococcal species at the time of inclusion. All of these patients were diagnosed with alcoholic liver cirrhosis, and four also had diabetes mellitus and progressed liver cirrhosis (Child B or C). Regarding the possibility of using antibiotics, only 2 patients had been hospitalized or exposed to antibiotics other than a member of the rifamycin group within the past 1 year. No one had a foreign body such as prosthetic joint, indwelling catheter, pacemaker, or prosthetic heart valve, which could cause a serious clinical condition from foreign body infection with rifampin-resistant staphylococcus species.

The mechanism of acquiring rifampin-resistant staphylococci in these patients is unclear. The mechanism of rifamycin-resistance through mutation of the *rpo*B gene has low risk for horizontal transmission compared with resistance encoded on mobile genetic elements (plasmid or transposon) [27]. These patients might have unclear recalls or have experienced exposure to other antibiotics in the rifamycin category. However, there was also a possibility that they harbored community- or hospital-acquired rifampin-resistant staphylococci. Although rifaximin resistance is not encoded on a mobile genetic element, rapid spread of such resistance is possible under the specific conditions of antibiotic over-use or inappropriate prescription [35], supported by a study of gene mutation-based ciprofloxacin resistance that spread rapidly after antibiotic treatment of MRSA in a nosocomial setting [36].

Although colonization with rifampin-resistant isolates did not result in a serious clinical status during the follow-up period in this study, it is still a cautious concern that broader use of rifaximin for mild disease, especially repeatedly or for long-term, could increase the possibility of significant infections with rifampin-resistant isolates in high-risk patients such as immunocompromised patients or those with a medical device implant. Therefore, further studies with long-term data are required to evaluate the clinical implication of the colonization of rifampin-resistant isolates after rifaximin use in immunocompromised patients and the risk of horizontal spread of rifampin resistance under the increased prescription of rifaximin with broad indications.

As this study was a pilot study, it has several limitations. First, there was inevitable variation in rifaximin treatment duration because this study was based in a real clinical setting; thus, the prescription schedule differed depending on patient clinical status. Also, a relatively small number of patients was enrolled, and we could not conclude exactly how long the resistant strain persisted after discontinuation of rifaximin treatment. However, this study is very valuable in terms of evaluating the incidence of staphylococcal cross-resistance to rifamycin after rifaximin treatment, as a result of clinical course in staphylococcus-colonized patients with immunocompromised condition for whom rifaximin drug use has been approved by the FDA.

In conclusion, rifampin-resistant staphylococcal strains can develop shortly after rifaximin treatment in cirrhotic immunocompromised patients. Although about the half of the resistant strains were no longer detected within about 4 months after rifaximin discontinuation without significant infectious events, the long-term drug influence on the clinical course in immunocompromised patients should be determined.

## Supporting information

S1 Fig Different kinds of staphylococcal isolates detected from hand (A) and perianal skin (B) of same patient in period 1. (A) is compatible with rifampin-sensitive *S*. *epidermidis*, and (B) is rifampin-resistant *S*. *aureus*.
(TIF)

S1 File Data of enrolled patients. This file contains clinical characteristics of enrolled patients used for this study. Information identifying the individual has been removed.
(XLSX)
