# Supplementary figures and images for: Emergence of rifampin-resistant staphylococci after rifaximin administration in cirrhotic patients
Source: PLoS One. 2017 Oct 5;12(10):e0186120. doi: 10.1371/journal.pone.0186120 (PMC5628927; doi:10.1371/journal.pone.0186120)

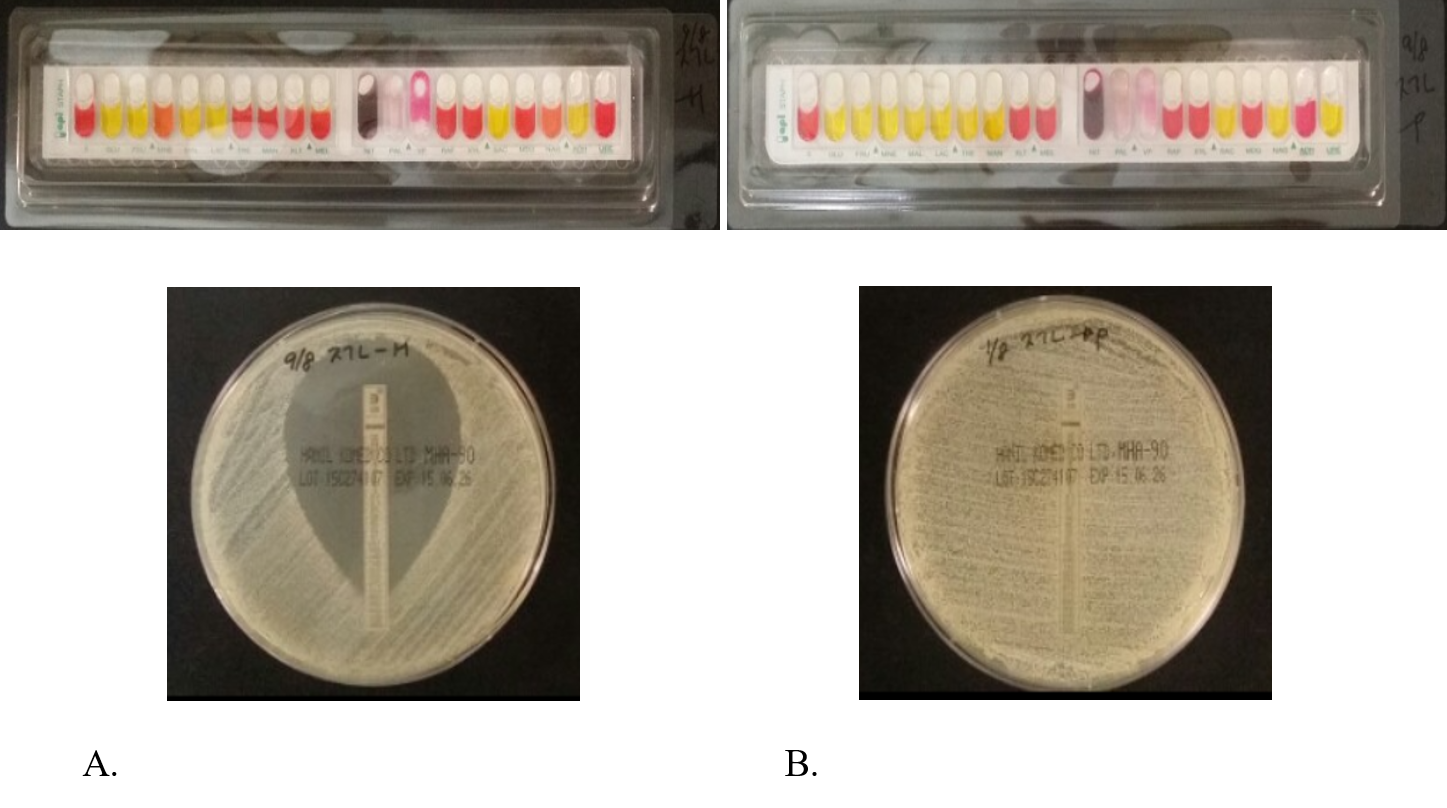

Supplement: S1 Fig — (A) is compatible with rifampin-sensitive S. epidermidis, and (B) is rifampin-resistant S. aureus. (TIF) [file pone.0186120.s001.tif]
